# Introspective Access or Retrospective Inference? Mind-Wandering Reports Are Shaped by Performance Feedback

**DOI:** 10.1177/09567976251349816

**Published:** 2025-06-30

**Authors:** Naya Polychroni, Mahiko Konishi, Isa Steinecker, Devin B. Terhune

**Affiliations:** 1 Department of Psychology, Goldsmiths, University of London; 2 Department of Experimental Psychology, University of Oxford; 3 Laboratoire de Sciences Cognitives et de Psycholinguistique, Department d’Etudes Cognitives, ENS, PSL University, EHESS, CNRS; 4 Bernstein Center for Computational Neuroscience (BCCN); 5 Department of Psychology, Institute of Psychiatry, Psychology & Neuroscience, King’s College London

**Keywords:** confidence, introspection, mind wandering, meta-awareness, vMRT

## Abstract

Most mind-wandering paradigms use self-reports following task performance, but the extent to which these reports are confounded by performance cues is unknown. In two experiments with adult human participants, we examined whether self-reports and confidence therein are influenced by performance indicators during visual metronome response tasks. In Experiment 1 (*N* = 40), sham feedback modulated reports independently of behavioral performance with participants more likely to report mind wandering after incorrect than correct sham feedback. In Experiment 2 (*N* = 111), we replicated this pattern using a more implicit manipulation of perceived performance—a surreptitious delay in the onset of response targets. Participants were more likely to report mind wandering after this delay than they were in control trials. In both experiments, confidence in on-task reports was lower when the corresponding indicator (falsely) implied poor performance. These findings suggest that mind-wandering reports and experiential state confidence are partly confounded by performance monitoring and have implications for experience-sampling methodologies.

## Introduction

A perennial challenge in the study of consciousness is how to grapple with the veracity of self-reports regarding subjective experience (Costall, 2006; Danziger, 1980). Nisbett and Wilson (1977) suggested that self-reports regarding mental processes do not arise from direct introspective access to the associated memories but instead rely on preconceptions pertaining to causal relationships between stimuli and responses.

There is now increased recognition that individuals often confabulate reasons to support their choices, which suggests limited access to higher-order mental processes (Johansson et al., 2005, 2006). Similarly, other lines of research have shown that, apart from mental processes, introspection regarding experiential states is also susceptible to contamination (Schooler & Schreiber, 2004).

One covert experiential state measured by the large majority of researchers via self-reports is the phenomenon of mind wandering (Smallwood & Schooler, 2015). Mind wandering is primarily assessed through experience sampling during sustained attention tasks in which participants are intermittently prompted to self-report whether they were mind wandering or not during the period immediately prior to the probe (Smallwood & Schooler, 2015). That is, the method employed to investigate the impact of mind wandering involves using participants’ retrospective self-reports to predict directly preceding behavior. In parallel, individuals vary greatly in the level of confidence they attribute to mind-wandering self-reports (Seli, Jonker, et al., 2015), with some participants reporting very low levels of confidence (Polychroni et al., 2022). This suggests that mind-wandering states are often imprecise and difficult to report. This represents a formidable challenge to the reliable measurement of mind-wandering states, the accuracy of which necessarily depends on our ability to introspect.

A wealth of evidence points to a close coupling between mind-wandering reports and behavioral performance (Randall et al., 2014). For example, mind-wandering reports are associated with increased error rates (e.g., McVay & Kane, 2009) and response time (RT) variability (e.g., Seli, Cheyne, & Smilek, 2013). This performance-report coupling is widely interpreted to reflect a deterioration in performance due to poorer attention and increased perceptual decoupling during mind-wandering states (Schooler et al., 2011). However, to date there has been no systematic consideration of the alternative hypothesis that this coupling is (partly) attributable to the use of performance cues to infer one’s experiential states. A potential confounding factor in performance-report coupling is that errors in sustained attention tasks are highly salient to participants (e.g., commission errors on no-go trials; Hawkins et al., 2022). In turn, performance monitoring may plausibly lead participants to attribute an error to mind wandering irrespective of their corresponding experiential state (see Head & Helton, 2016). Previous research has suggested that performance monitoring (feedback error–related negativity) is attenuated during mind wandering (Kam et al., 2012), but this does not mean that performance monitoring is completely disrupted. Accordingly, we propose that participants retrospectively infer experiential states from an admixture of available evidence, including both introspective cues (metacognitive awareness) and external contextual information (performance cues).

In two experiments, we investigated the impact of performance monitoring on self-reported mind wandering by manipulating performance cues. Participants completed the visual metronome response task (vMRT; Laflamme et al., 2018) while they were intermittently probed to report experiential states (on task vs. mind wandering), meta-awareness (aware vs. unaware mind wandering), and confidence in these reports. We manipulated perceived performance with sham (random) feedback (Experiment 1) and by introducing a surreptitious delay in the onset of response targets (Experiment 2). On the basis of our inference hypothesis, we expected that participants would be more likely to self-report mind wandering after wrongly perceiving that they had made an error. We further expected that mind-wandering reports after misperceived errors would be rated with higher confidence than those after misperceived accurate responses; we expected the reverse for on-task reports. Finally, we explored whether these effects would also hold for meta-awareness of mind wandering. We expected that participants would report more unaware mind wandering after misperceived errors (similar to mind wandering more broadly), or, alternatively, that they would report more aware mind wandering after such errors because of corroboration of performance decrements.

## Research Transparency Statement

### General disclosures

**Conflicts of interest:** All authors declare no conflicts of interest. **Funding:** This research was supported by funding from Goldsmiths, University of London to N. Polychroni and a Biotechnology and Biological Sciences Research Council grant (No. BB/R01583X/1) to D. B. Terhune. **Artificial intelligence:** No artificial-intelligence-assisted technologies were used in this research or the creation of this article. **Ethics:** This research received approval from a local ethics board.

### Experiment 1 disclosures

**Preregistration:** No aspects of the study were preregistered. **Materials:** All study materials are publicly available (https://osf.io/n3zhx). **Data:** All primary data are publicly available (https://osf.io/jk5gw). **Analysis scripts:** All analysis scripts are publicly available (https://osf.io/x4ptb). **Computational reproducibility:** The computational reproducibility of the results has been independently confirmed by the journal’s STAR team.

### Experiment 2 disclosures

**Preregistration:** No aspects of the study were preregistered. **Materials:** All study materials are publicly available (https://app.gorilla.sc/openmaterials/792863). **Data:** All primary data are publicly available (https://osf.io/sra98). **Analysis scripts:** All analysis scripts are publicly available (https://osf.io/56rqk). **Computational reproducibility:** The computational reproducibility of the results has been independently confirmed by the journal’s STAR team.

## Experiment 1

### Method

#### Participants

Sample size was estimated a priori with the goal to run 40 participants, which enabled detecting effect sizes of Cohen’s *d* ≥ 0.45 (two-tailed α = .05, 1 − β = 0.80). Participants were recruited via advertisements at Goldsmiths, University of London. Forty healthy adults with normal or corrected-to-normal vision provided informed written consent to participate and were compensated £7 per hour (25 females, age range = 19–37 years, *M*_age_ = 27.05, *SD* = 5.01). The data were not examined until collection was completed to avoid optional stopping. The study was approved by the Research Ethics Committee of the Department of Psychology at Goldsmiths, University of London.

#### Materials

**Fig. 1.**
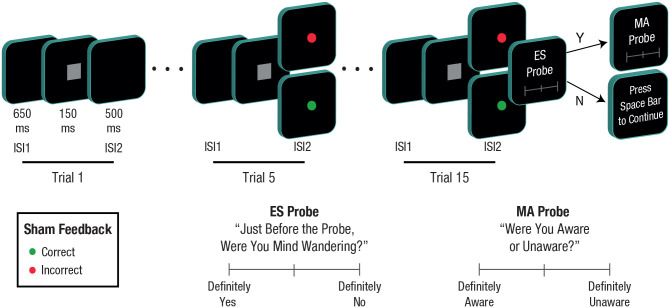
Visual representation of the visual metronome response task (vMRT) in Experiment 1. See text for further details. ES = experiential state; MA = meta-awareness; ISI = interstimulus interval.

Participants completed the vMRT, a standardized and well-validated measure of mind wandering (Laflamme et al., 2018; Fig. 1). They were presented with a visual stimulus (square) at a constant rhythm and responded by pressing the space bar in synchrony with it. Each trial consisted of a blank black interstimulus interval, or ISI1 (650 ms), followed by a centrally-presented gray square (3.5 cm^2^; 150 ms), and a second blank ISI, called ISI2 (500 ms). Sham feedback, in the form of a centrally presented colored circle (green = correct, red = incorrect, diameter = 0.8 cm) was presented on every fifth trial during the last 100 ms of ISI2. Sham feedback was pseudorandomly distributed within each block (50% correct, 50% incorrect) irrespective of actual performance, such that all participants were told they were doing well in half the trials of each block and poorly in the other half. Experiential-state probes were presented after either 15 or 20 trials (counterbalanced and in random order): “Just before the probe, were you mind wandering?” Participants also rated their confidence in their experiential-state report using a visual analogue scale ranging from 0 to 100, with anchors ranging from *definitely yes* to *definitely no*. If participants responded affirmatively, they next reported their meta-awareness of mind wandering and their confidence—“Were you aware or unaware?”—on a scale with anchors *definitely aware* and *definitely unaware*.

#### Procedure

Mind wandering was defined to participants as any thoughts that are not related to the current task and are usually internally focused (Smallwood & Schooler, 2015; Smallwood et al., 2008); examples of mind wandering were presented (e.g., thoughts about past events, friends, or significant others). *Aware mind wandering* was defined as a state in which the mind wanders and participants are aware all along that it is doing so; *unaware mind wandering* was defined as a state in which the mind wanders but participants are unaware that it is doing so until they catch themselves (Smallwood et al., 2008). Next, vMRT instructions directed participants to press the space bar in synchrony with the onset of the square so that they press the space bar exactly when each square is presented—that is, trying to synchronize with the metronome rather than reacting to it (Laflamme et al., 2018). Participants were subsequently given instructions regarding the introduction of performance feedback and were informed that the feedback (appearing on every fifth trial) reflected a cumulative measure of their performance in the five preceding trials.

After the instructions, participants completed a practice block of 50 trials without feedback and with three intermittent probes, after which they passively watched a demonstration of 10 trials with performance feedback on the monitor. Next, participants completed six blocks of 210 trials interspersed with 12 experiential-state probes (i.e., 1,260 trials and 72 probes in total). Sham feedback was presented in 252 trials, 72 of which preceded a probe. Blocks took approximately 8 min to complete, with the entire experiment lasting approximately 1 to 1.25 hr. The experiment was programmed and implemented in MATLAB (2018a, The MathWorks, Natick, MA), using the Psychophysics Toolbox extensions (Kleiner et al., 2007). After finishing the task, participants completed (using pen and paper) a posttask questionnaire regarding their awareness of the manipulation (see the Supplemental Material available online).

As in previous studies (Laflamme et al., 2018), we planned to exclude participants with omission-error rates greater than 10%, but no one’s data exceeded this threshold (*M* = 0.74% *SD* = 1.31, range = 0–7.1%). Note that these measures were computed for 1,189 trials; we removed the first trial after each probe because the square appeared after 500 ms in this trial. Rhythmic response times (RRTs) were calculated as the relative time difference between the response and stimulus onset. RRT variance (RRTV) was computed for the five trials preceding each experiential-state probe (Seli, Cheyne, & Smilek, 2013). RRTV data were positively skewed (skewness = 3.16) and were transformed using a natural logarithm (Laflamme et al., 2018), which successfully reduced skewness (−1.26). Twenty-five sequences (0.87%) that contained more than one omission error were excluded, resulting in a total of 2,855 trials across the 40 participants (trials per participant: *M* = 71.38, *SD* = 1.53, range = 64–72). Three participants who did not report any unaware mind wandering were excluded from the meta-awareness analyses, resulting in 1,360 trials (trials per participant: *M* = 36.76, *SD* = 14.61, range = 7–68) for these analyses. No outliers (between-participants: *M* ± 3 *SD*) were detected.

Data were analyzed using mixed-effects models (Bolker et al., 2009; Meteyard & Davies, 2020). We examined whether experiential-state reports (0 = on task, 1 = mind wandering) and meta-awareness reports (0 = aware, 1 = unaware) varied as a function of sham feedback (0 = correct, 1 = incorrect) and RRTV prior to probes in two generalized linear mixed-effects models. Next, two linear mixed-effects models were used to predict confidence in experiential-state and meta-awareness reports using Sham Feedback × Experiential State and Sham Feedback × Meta-Awareness as predictors, respectively. For completeness, all models were extended to include RRTV and RRTV interactions with the main factors (see the Supplemental Results section in the Supplemental Material).

The fixed factors were added to each model after specifying the random effect structure. Random effects were added on the basis of model fit (likelihood ratio test) through forward selection (Bates et al., 2015; Meteyard & Davies, 2020). We began by comparing a by-participant random intercept model to two models, each of which included a random slope for one of the fixed factors. If only one random slope improved model fit, we selected that as the final model. If both random slopes improved the fit, we compared the best model from the previous step to a model with both random slopes. If this model did not offer a better fit, we retained the best model from the previous step. If the model with both random slopes provided a better fit, we compared it to a model that also included a random slope for the interaction. However, in all cases, the latter models did not result in a better fit. The final model for experiential-state reports included random slopes of both sham feedback and RRTV, whereas the model for meta-awareness reports retained only the random intercept. The final models for confidence in experiential-state and meta-awareness reports included random slopes for sham feedback and experiential state, and random slope for meta-awareness, respectively.

To test the significance of effects, we used a likelihood ratio test comparing models (Barr et al., 2013) that differed only with respect to the fixed effect of interest and that shared the same random-effects structure (see the Supplemental Results section in the Supplemental Material for detailed model comparisons). Therefore, for significant effects we additionally report χ^2^ statistics. All analyses were implemented in MATLAB (2018a).

### Results

#### Summary statistics

Participants reported mind wandering on approximately half of experiential-state probes, *M* = 51.01%, *SD* = 20.97% (range = 9.72–94.44), rates that are similar to previous studies (Seli, Cheyne, et al., 2015). Among self-reported mind-wandering states, participants reported aware mind wandering on nearly two thirds of the probes, *M* = 62.14%, *SD* = 26.75%.

Responses on the posttask questionnaire indicated that only 9 out of 40 participants (22.5%) reported that the feedback was unusual when asked whether they had noticed anything unusual about the task. When directly asked about the feedback, 24 participants (60%) reported that they noticed something unusual about the feedback, but only 2 participants stated that they thought the feedback was random. A number of participants (26, or 65%) reported that they found the feedback helpful. Overall, participants reported paying attention to the feedback over three quarters of the time, *M* = 79.65%, *SD* = 16.90% (range = 25–100) and tended to consider the feedback accurate, *M* = 68.93%, *SD* = 19.44% (range = 25–100). Interestingly, participants found correct feedback to be accurate more often, *M* = 74.88%, *SD* = 20.27%, than incorrect feedback, *M* = 63.10%, *SD* = 21.88%, *t*(39) = 3.23, *p* = .003, *g* = .55. These results indicate that the feedback was generally believed by most participants, suggesting that they did not infer that the feedback was not representative of their performance.

#### Predicting experiential-state and meta-awareness reports from sham feedback

**Fig. 2.**
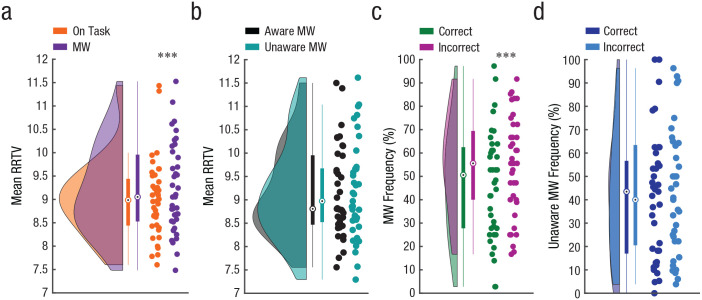
Performance, mind wandering, and meta-awareness of mind wandering in the visual metronome response task (vMRT) in Experiment 1 (a–c: *N* = 40; b–d: *N* = 37). Mean rhythmic response time variance (RRTV) is shown as a function of (a) experiential-state reports and (b) meta-awareness reports; self-reports are shown as a function of sham feedback for (c) mind-wandering frequency and (d) unaware mind-wandering frequency. Boxplots denote the median and interquartile range, and whiskers extend to the most extreme data points not considered outliers. Marginal plots reflect kernel density plots. ***p < .001.

We first tested the prediction that experiential-state reports are shaped partly by performance cues independently of actual behavioral response patterns. First, we investigated whether experiential-state reports varied as a function of behavioral performance (RRTV) and sham feedback prior to experiential-state probes (Table 1 and Fig. 2). RRTV positively predicted experiential-state reports: Mind-wandering reports increased with greater RRTV (likelihood ratio test: χ^2^(1) = 8.33, *p* = .004), replicating previous findings (Laflamme et al., 2018; Seli, Jonker, et al., 2015). In addition, as predicted by our inference hypothesis, mind-wandering frequency was higher when participants were given incorrect sham feedback,^1^ χ^2^(1) = 12.55, *p* < .001 (Table 1). Next, we examined whether these effects were present for meta-awareness reports. Sham feedback did not significantly predict meta-awareness reports, *B* = 0.10, *SE* = 0.13, 95% confidence interval (CI) = [−0.15, 0.35], *p* = .45. Similarly, RRTV was not a significant predictor of meta-awareness reports, *B* = −0.01, *SE* = 0.05, 95% CI = [−0.11, 0.08], *p* = .80 (Seli et al., 2013).

**Table 1.** Generalized Linear Mixed-Effects Model of Experiential State Reports in Experiment 1 (*N* = 40)

ES ~ 1 + SF + RRTV + (1|pID) + (SF − 1|pID) + (RRTV − 1|pID)						
Variable	*B*	*SE*	*t*	*df*	*p*	95% CIs
Intercept	−1.06	0.31	−3.44	2852	< .001	[−1.67, −.46]
Sham feedback	0.38	0.10	3.83	2852	< .001	[.18, .57]
RRTV	0.10	0.03	2.93	2852	.003	[.03, 17]
Linear mixed-effects model of ES report confidence (*N* = 40)						
C_ES_~ 1 + SF × ES + (1|pID) + (SF − 1|pID) + (ES − 1|pID)						
Intercept	71.77	2.81	25.51	2851	< .001	[66.26, 77.29]
Sham feedback	−6.07	1.53	−3.96	2851	< .001	[−9.07, −3.06]
ES	−11.94	3.00	−3.98	2851	< .001	[−17.83, −6.05]
SF × ES	7.92	1.95	4.07	2851	< .001	[4.11, 11.74]

Note: C_ES_ = ES report confidence; ES = experiential state (0 = on task, 1 = MW); SF = sham feedback (0 = correct, 1 = incorrect); RRTV = rhythmic response-time variance; pID = participant ID; CI = confidence interval.

#### Predicting confidence from sham feedback and self-reports

**Fig. 3.**
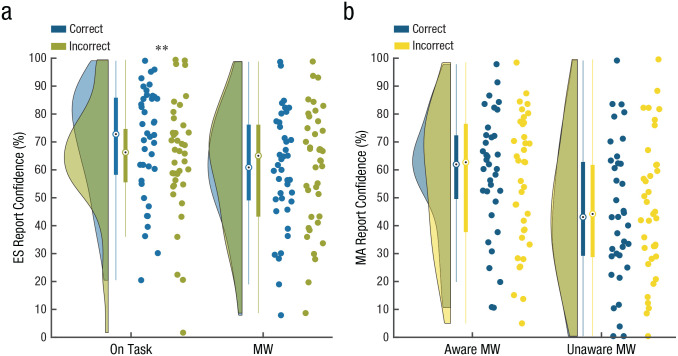
Confidence ratings (%) as a function of sham feedback and (a) experiential-state reports (*N* = 40) and (b) meta-awareness reports (*N* = 37) in the visual metronome response task (vMRT) in Experiment 1. Boxplots denote the median and interquartile range, and whiskers extend to the most extreme data points not considered outliers. Marginal plots reflect kernel density plots. ES = experiential state; MW = mind wandering. **p < .01.

Our central prediction regarding confidence in experiential-state reports was that mind-wandering reports after negative sham feedback would be rated with higher confidence than those after positive sham feedback and that the inverse would hold for on-task reports. Accordingly, we expected that the sham feedback effect would be moderated by experiential-state (ES) report (Sham Feedback × ES interaction). The results of the model confirmed this prediction (Table 1 and Fig. 3): ES (likelihood ratio test: χ^2^(1) = 13.56, *p* < .001), sham feedback, χ^2^(1) = 13.29, *p* < .001, and Sham Feedback × ES, χ^2^(1) = 15.32, *p* < .001, all significantly predicted confidence. Subsidiary analyses showed that confidence in on-task reports was significantly lower for incorrect than correct sham feedback, *B* = −6.03, *SE* = 1.83, 95% CI = [−9.62, −2.43], likelihood ratio test: χ^2^(1) = 9.35, *p* = .002, whereas the effect of sham feedback was not significant for mind-wandering reports, *B* = 2.08, *SE* = 1.64, 95% CI = [−1.13, 5.29], *p* = .20.

Our final analysis explored whether meta-awareness (MA) report confidence would be similarly predicted by the Sham Feedback × MA interaction (Fig. 3). Neither sham feedback, *B* = −0.58, *SE* = 1.81, 95% CI = [−4.13, 2.96], *p* = .75, nor the Sham Feedback × MA interaction, *B* = 2.93, *SE* = 2.87, 95% CI = [−2.70, 8.56], *p* = .31, were significant predictors of confidence and were thus removed from the model. However, meta-awareness report confidence was significantly predicted by meta-awareness reports: participants were more confident in aware than unaware mind-wandering reports, *B* = −12.90, *SE* = 4.34, 95% CI = [−21.41, −4.38], likelihood ratio test: χ^2^(1) = 7.19, *p* = .007.

### Discussion

In support of our hypothesis that experiential-state reports are partly shaped by performance cues, mind-wandering rates were higher after perceived errors than perceived correct responses. Importantly, sham feedback improved upon a model of mind-wandering rates driven by behavioral performance. This effect was also partly reflected in confidence in experiential states: confidence in on-task reports was greater after perceived correct responses than perceived errors. These findings suggest that, rather than directly accessing experiential states, we partly infer them on the basis of performance cues. Nevertheless, explicit feedback may have affected attentional control (Adam & Vogel, 2016; Bejjani et al., 2020; see also deBettencourt et al., 2015) or may have biased participants’ reports by triggering participants to respond in accordance with task demands (Forster et al., 2022).

## Experiment 2

In order to circumvent the potential limitations of Experiment 1, we aimed to conceptually replicate our results using a more implicit manipulation of perceived performance. Participants completed the vMRT with the same intermittent experiential-state probes. Rather than manipulating feedback, we introduced a surreptitious delay for a subset of response targets. Drawing on our inference hypothesis that mind-wandering reports are partly influenced by performance monitoring, we expected that target delays would produce a prediction error (Ito et al., 2022) so that participants would infer that they had made an error (premature response) and, in turn, attribute this to mind wandering.

### Method

#### Participants

A sample size of 120 participants was prespecified, which enabled us to detect effect sizes of Cohen’s *d* ≥ 0.26 (two-tailed α = .05, 1 − β = 0.80). The data were not examined until data collection was completed to avoid optional stopping. Participants were recruited via the Prolific platform (https://www.prolific.co), and they completed the task online via Gorilla (www.gorilla.sc) (Anwyl-Irvine et al., 2020). All participants were compensated £5 per hour. Three participants that did not report normal or corrected-to-normal vision were excluded, resulting in a final sample of 117 participants (77 females, 39 males, 1 nonbinary, age range = 18–59 years, *M*_age_ = 30.77, *SD* = 10.23). The study was approved by the Research Ethics Committee of the Department of Psychology at Goldsmiths, University of London.

#### Materials

**Fig. 4.**
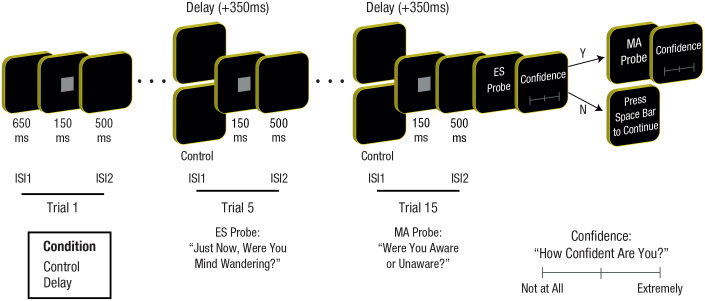
Visual representation of the vMRT in Experiment 2. See the text for further details. Y = Yes, N = No, ES = experiential state; MA = meta-awareness.

The vMRT was modified to include two trial types (control and delay; Fig. 4). Control trials (the standard version of vMRT) consisted of a blank black screen (ISI1; 650 ms), followed by a centrally presented gray square as the target (3.5 cm^2^; presented for 150 ms), and a second blank ISI (ISI2; 500 ms; Laflamme et al., 2018). In delay trials, a 350-ms delay was inserted prior to targets after participants’ responses. If participants did not respond within 900 ms of ISI1 onset, the delay was skipped to minimize the transparency of the trial manipulation. The target-delay manipulation was included on every fifth trial (50% control, 50% delay). Experience sampling using probes was the same as in Experiment 1.

### Procedure

The instructions were identical to those of Experiment 1. Participants were falsely informed that the rhythm of the target presentation would be constant throughout the task. They were further requested to complete the task using their right index finger and to click only once per trial. Subsequently, participants completed a practice block of 35 trials with two embedded probes. In the main experimental phase, participants completed eight blocks of 140 trials (1,120 trials in total) with 64 experiential-state probes (eight per block). Eight miniblocks comprised each block, with four miniblocks consisting of 15 trials and four miniblocks consisting of 20 trials. All miniblocks ended with an experiential-state probe. Each fifth trial was either a control or a delay trial, so that half the miniblocks (within each block and across the task) ended in a delay and half ended in a control trial. Therefore, across the 1,120 trials there were 228 trials indexed as either a control or a delay trial, 64 of which (32 delay, 32 control trials) immediately preceded an experiential-state probe. Block order was the same for all participants, but the order of miniblocks within each block was random. After completing the task, participants completed a posttask questionnaire regarding awareness of the manipulation (see the Supplemental Material).

### Statistical analyses

Six participants were excluded because of insufficient self-reported mind wandering (< 5 probes; *n* = 3) or high response omission rates (> 10%; Laflamme et al., 2018; *n* = 3), resulting in a final sample of 111 participants with a mean of 1.83% omissions (*SD* = 1.73). In the case of two responses on an individual trial (*M* = 2.66%, *SD* = 8.29%), only the first response was considered. RRTV was computed for each sequence of five trials preceding probes. RRTV was also computed for each sequence, after excluding the trial preceding the probe, to remove a potential influence of target delays on the final trial RT. Thirty-nine sequences that contained more than one omission were excluded, resulting in 7,065 trials (per participant: *M* = 63.65, *SD* = 1.18, range = 54–64). In both cases, RRTV data were positively skewed (skewness = 5.53 and 5.70, respectively) and successfully transformed using a natural-logarithm transform (−.25 and −.49, respectively; Laflamme et al., 2018). Eight participants who did not report any unaware mind wandering were excluded from meta-awareness analyses, resulting in 2,961 trials (*M* = 28.75, *SD* = 13.93, range = 5–58, *N* = 103).

The data were analyzed in the same manner as in Experiment 1. Two separate generalized linear mixed-effects models were used to predict experiential-state reports (0 = on task, 1 = mind wandering) and meta-awareness reports (0 = aware, 1 = unaware) using RRTV and condition (0 = control, 1 = delay) as predictors. Next, two linear mixed-effects models were used to predict experiential-state and meta-awareness report confidence with Condition × ES and Condition × MA, respectively, as predictors. These factors were added after establishing the random-effect structure for each model following forward selection as in Experiment 1. The final models for both experiential-state and meta-awareness reports, as well as meta-awareness report confidence, retained only the random intercept for each participant. The final model for confidence in experiential-state reports additionally included a random slope for experiential state. As in Experiment 1, models were extended to include RRTV and RRTV interactions with the main factors (see the Supplemental Results section in the Supplemental Material). In addition, a series of sensitivity analyses were conducted in order to replicate the primary results after excluding participants with atypical response patterns (see the Supplemental Results).

### Results

#### Data summary

Participants reported mind wandering on almost half of the probes in the task, *M* = 43.89%, *SD* = 22.46% (range = 7.81–92.19), a small decrease relative to Experiment 1 (51%). Among self-reported mind-wandering trials, participants reported aware mind wandering on almost two thirds of the probes, *M* = 65.73%, *SD* = 22.76% (range = 13.79–100). Responses to the posttask questionnaire indicated that only 29 of 111 participants (26%) reported that they observed something unusual about the task; 20 of them (18%) specifically referred to the atypical target rhythm rate. When directly asked about the rate of the rhythm, 91 participants (82%) reported they noticed variability in the rate of the square’s appearance. This suggests that although many participants noticed a difference in the rhythm, relatively few directly referred to it when asked generally about the task.

#### Evaluating the effect of target delays on performance (RRTV)

It is plausible that target delays would impact performance in such a way that participants would take longer to respond on trials that included a target delay, especially if participants became accustomed to reacting to the square rather than synchronizing with the rhythm. Indeed, half (50%) of participants responded on average after the onset of the stimulus, and RRTV was higher in sequences that included a delay trial, *B* = 0.15, *SE* = 0.04, 95% CI = [0.07, 0.23], likelihood ratio test: χ^2^(1) = 12.46, *p* < .001, suggesting that performance in the trial before the probe might be associated with compensatory behavior from the change in the rhythm. To address this, we considered whether RRTV could be predicted from condition when the final trial (i.e., the one potentially influenced by compensatory behaviors) was omitted. This revised RRTV measure was not significantly predicted by condition, *B* = −0.02, *SE* = 0.03, 95% CI = [−0.09, 0.04], *p* = .45, as would be expected given that the target-delay manipulation occurred only afterward. In order to minimize the impact of this confound, we used the revised four-trial RRTV measure in all subsequent analyses. Analyses including the conventional five-trial measure yielded comparable results (see the Supplemental Results in the Supplemental Material).

#### Predicting experiential state and meta-awareness reports from target delays and performance

**Fig. 5.**
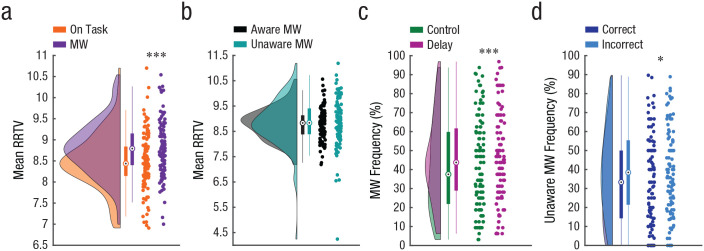
Performance, mind wandering, and meta-awareness (MA) of mind wandering in the visual metronome response task (vMRT) in Experiment 2 (in a and c, *N* = 111; in b and d, *N* = 103). In (a) we show mean rhythmic response time variance (RRTV) as a function of experiential state (ES) reports; in (b), we show mean RRTV as a function of MA reports. In (c) are illustrated self-reports as a function of condition for mind-wandering frequency; in (d) are illustrated self-reports as a function of condition for unaware mind-wandering frequency. Boxplots denote the median and interquartile range, and whiskers extend to the most extreme data points not considered outliers. Marginal plots reflect kernel density plots. *p < .05. ***p < .001.

We first tested the prediction that participants would report mind wandering more frequently after delay than control trials. The results of the model (Table 2) showed that RRTV was higher during mind wandering (likelihood ratio test: χ^2^(1) = 56.13, *p* < .001; see Fig. 5), thus replicating Experiment 1 and previous results (Laflamme et al., 2018). Critically, the addition of a target delay influenced participants’ reports: They reported mind wandering more frequently after delay than after control trials, χ^2^(1) = 14.38, *p* < .001.

**Table 2.** Generalized Linear Mixed-Effects Model of Experiential-State Reports in Experiment 2 (*N* = 111)

ES ~ 1 + Condition + RRTV + (1|pID)						
Variable	*B*	*SE*	*t*	*df*	*p*	95% CIs
Intercept	−1.85	0.21	−9.03	7062	< .001	[−2.25, −1.45]
Condition	0.20	0.05	3.82	7062	< .001	[.10, .31]
RRTV	0.17	0.02	8.39	7062	< .001	[.13, .21]
Linear mixed-effects model for confidence in ES reports (C_ES_; *N* = 111)						
C_ES_~ 1 + Condition + Condition × ES + (1|pID) + (ES-1|pID)						
Intercept	74.05	1.57	47.19	7062	< .001	[70.97, 77.13]
Condition	−1.32	0.47	−2.84	7062	.005	[−2.24, −0.41]
Condition × ES	2.12	0.71	3.00	7062	.003	[0.73, 3.51]

Note: C_ES_ = ES report confidence; ES = experiential state (0 = on task, 1 = MW); condition: 0 = control, 1 = delay; RRTV = rhythmic response-times variance; pID = participant ID; CI = confidence interval.

Next, consistent with Experiment 1, we found that RRTV did not significantly predict meta-awareness reports (Seli, Cheyne, & Smilek, 2013), *B* = 0.03, *SE* = 0.03, 95% CI = [−0.04, 0.09], *p* = .42 (Fig. 5). By contrast, the condition effect was significant, *B* = 0.17, *SE* = 0.08, 95% CI = [.01, .34], likelihood ratio test: χ^2^(1) = 4.08, *p* = .043, with participants more frequently reporting unaware mind wandering after a delay. However, this effect should be interpreted with caution because it was not observed in Experiment 1 and was not significant in a reduced sample (see the Supplemental Results in the Supplemental Material).

#### Predicting confidence from target delays and self-reports

**Fig. 6.**
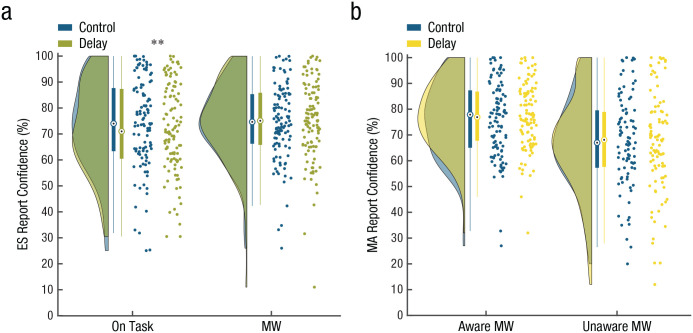
Confidence ratings (%) as a function of condition and (a) experiential state reports (ES; *N* = 111) and (b) meta-awareness reports (MA; *N* = 103) in the visual metronome response task (vMRT) in Experiment 2. Boxplots denote the median and interquartile range, and whiskers extend to the most extreme data points not considered outliers. Marginal plots reflect kernel density plots. MW = mind wandering. **p < .01.

We next assessed the prediction that the impact of condition on experiential-state report confidence would be moderated by experiential-state report (Table 2). Condition was significant (likelihood ratio test: χ^2^(1) = 8.05, *p* = .005), reflecting lower confidence across states following target delays. However, contrary to Experiment 1, confidence was not significantly predicted by experiential-state report, *B* = 0.61, *SE* = 1.41, 95% CI = [−2.14, 3.37], *p* = .66. Consistent with our prediction, the Condition × ES interaction was significant, χ^2^(1) = 8.97, *p* = .003: Mind-wandering reports were rated with higher confidence when probes were preceded by delay trials, whereas the opposite was observed for on-task reports (Fig. 6). Subsidiary analyses showed that condition significantly predicted confidence among on-task reports, *B* = −1.32, *SE* = 0.47, 95% CI = [−2.25, −0.39], likelihood ratio test: χ^2^(1) = 7.72, *p* = .005, but not among mind-wandering reports, *B* = 0.78, *SE* = 0.52, 95% CI = [−0.24, 1.79], *p* = .13. Our final analysis examined whether meta-awareness report confidence would be predicted by the Condition × MA interaction (Fig. 6). Both condition, *B* = 0.78, *SE* = 0.50, 95% CI = [−0.20, 1.77], *p* = .12, and Condition × MA, *B* = −1.67, *SE* = 1.04, 95% CI = [−3.71, 0.37], *p* = .11, were not significant. Meta-awareness was a significant predictor, *B* = −9.01, *SE* = 1.35, 95% CI = [−11.66, −6.36], likelihood ratio test: χ^2^(1) = 37.40, *p* < .001: participants were more confident in reporting aware than unaware mind wandering.

### Discussion

Consistent with our inference hypothesis, participants were more likely to report mind wandering when there was a target delay relative to when there was no such delay. Moreover, target delays modulated confidence such that participants were less confident in on-task reports after a delay, than a control, trial. These results suggest that target delays led participants to infer that they had responded prematurely because of mind wandering, thereby artificially increasing their mind-wandering rates and reducing their confidence in on-task reports. Importantly, these effects were observed independently of behavioral performance, which was an independent predictor of experiential-state reports.

## General Discussion

Through two experiments, we provide evidence that performance monitoring shapes both self-reports regarding experiential states and confidence therein. Using sham feedback (Experiment 1) and surreptitious response target delays (Experiment 2) as performance cues, we observed that participants were more likely to report mind wandering under conditions when they inferred that they had made an error irrespective of behavioral performance. These manipulations also affected confidence: Confidence in on-task reports was lower after misperceived errors. The impact of performance cues on meta-awareness of mind wandering was more equivocal: Sham feedback did not significantly affect meta-awareness reports (Experiment 1), whereas participants reported a weak tendency toward more unaware mind wandering after target delays (Experiment 2). Cumulatively, these results suggest that experiential-state reports are confounded by performance cues.

The present results suggest that mind-wandering reports reflect inferences that integrate performance monitoring with metacognitive appraisals of experiential states. Participants may expect that they are more likely to perform poorly when they are mind wandering on the basis of folk beliefs regarding mind wandering, learned associations between mind wandering and task performance in daily life, and expectations related to contextual factors in a task. Experimental instructions and experimentally presented definitions of mind wandering may also play a role. This aligns with theoretical accounts of distortions in the process of rerepresenting experience (translation dissociations) via substitution—that is, individuals accessing expectations about an experience rather than the experience itself (Schooler & Schreiber, 2004). Interestingly, failure to observe a robust effect of performance cues on meta-awareness aligns with this interpretation. In other words, it may reflect the possibility that individuals do not form precise predictions regarding the relationship between meta-awareness and performance and thus do not reliably use performance to inform these reports. Indeed, prototypical definitions of meta-awareness (Smallwood et al., 2007) do not imply a relationship between meta-awareness and task performance, whereas the two are strongly linked in most mind-wandering definitions (Smallwood & Schooler, 2015). Our findings on confidence suggest that self-reports are validated when (perceived) performance is congruent with predictions, resulting in greater confidence when performance cues align with self-reports. Alternatively, these effects may be due to response biases in which reports are made on the basis of behavioral cues because of low effort or motivation (Krosnick, 1999; Weinstein, 2018).

Our findings have broad implications for the use of self-report methods to index mind wandering. We propose that the observed effects can be understood within the predictive processing framework wherein mind-wandering reports can be conceptualized as a form of Bayesian inference. This framework theorizes that experience reflects an inference on the basis of an admixture of top-down priors and sensory evidence, the impact of which is driven by precision weighting (Clark, 2013; Friston, 2010). We suggest that performance cues function as (precise) priors (i.e., probabilistic expectations) that are weighted against introspective evidence of one’s experiential state (likelihood) in order to infer experiential states (posteriors). In line with this suggestion, recent evidence has shown that reported inattention without remembering the content of thoughts is associated with poor performance, whereas remembered mind-wandering episodes are not (Mathews et al., 2024). This may be due to a stronger weighting of performance cues when introspective cues are not available.

The observed effects challenge the prevailing assumption regarding the causal chain between behavior and self-reports, namely that fluctuations in experiential states, as indexed by self-reports, underlie performance differences. As in our study, mind-wandering reports have been consistently related to RT variability (Laflamme et al., 2018), as well as impaired performance (Mooneyham & Schooler, 2013). However, the present results point to a more nuanced relationship between performance and mind wandering: although mind wandering reliably impairs performance, the perception of poorer performance also seems to trigger self-reported mind wandering through the inference process described above. Importantly, these effects will plausibly be most pronounced in tasks in which it is easy to monitor one’s performance, such as widely used sustained-attention tasks (Hawkins et al., 2022), and they may be modulated by task parameters, such as difficulty, which is a salient factor in theoretical accounts of mind wandering (Seli et al., 2018). Therefore, caution is required when prompting participants’ self-reports retrospectively to a coincident performance marker. These effects could be reduced by avoiding explicit behavioral indicators prior to experiential-state probes and by modifying instructions to participants to minimize demand-characteristics effects (Forster et al., 2022). Nevertheless, further research is needed to assess whether the effects generalize to other tasks.

The results of this research should not be interpreted as reflecting a sweeping limitation of self-reports: self-reported mind wandering is associated with distinct brain patterns compared to on-task reports (Christoff et al., 2009; Kam et al., 2022) and predicts other measures, including eye movements (Faber et al., 2018) and pupil dilation (Konishi et al., 2017), which are unlikely to be related to the observed effects. It is important to note that our data do not show that reports based on inferences from behavioral cues are inherently incorrect per se (although sometimes they will be). Instead, this form of inference could be an effective way of gaining access to experiential states and redirecting attention to one’s current task, especially when introspective access is compromised. Rather than dismissing introspection, future research should seek out further methodological improvements in the measurement of mind wandering in order to obtain more precise estimates of experiential states.

A potential limitation of this work is that our main interpretation rests on the assumption that participants believe that mind-wandering states are characterized by poorer performance on the basis of prior experience and the experimental context. Future work should aim to more directly assess beliefs regarding the relationship between mind wandering and performance (Irving et al., 2020) and examine to what extent these beliefs moderate the effects observed here. All things considered, the present findings suggest that these confounds warrant further empirical attention, including extensions to other subjective phenomena (Barrett & Simmons, 2015).

In summary, in two experiments, participants were more likely to report mind wandering when contextual cues signified that they had made an error. These effects were independent of behavioral performance and suggest that mind-wandering reports are not solely derived from introspective access to experiential states. Rather, we propose that mind-wandering reports, and confidence therein, reflect an inferential process in which contextual cues and introspective evidence are integrated to allow experiential-state estimates. These results highlight the confounding effect of performance monitoring in the assessment of mind wandering and have implications for the measurement of experiential states more broadly.

## Supplemental Material

sj-pdf-1-pss-10.1177_09567976251349816 – Supplemental material for Introspective Access or Retrospective Inference? Mind-Wandering Reports Are Shaped by Performance Feedback Supplemental material, sj-pdf-1-pss-10.1177_09567976251349816 for Introspective Access or Retrospective Inference? Mind-Wandering Reports Are Shaped by Performance Feedback by Naya Polychroni, Mahiko Konishi, Isa Steinecker and Devin B. Terhune in Psychological Science

## Transparency

*Action Editor:* Ulrike Hahn

*Editor:* Simine Vazire

*Author Contributions*

**Naya Polychroni:** Conceptualization; Data curation; Formal analysis; Investigation; Methodology; Project administration; Software; Validation; Visualization; Writing – original draft; Writing – review & editing.

**Mahiko Konishi:** Methodology; Writing – review & editing.

**Isa Steinecker:** Methodology; Writing – review & editing.

**Devin B. Terhune:** Conceptualization; Data curation; Methodology; Project administration; Software; Supervision; Validation; Writing – review & editing.

**Artificial Intelligence:** No artificial intelligence assisted technologies were used in this research or the creation of this article.

**Ethics:** This research received approval from a local ethics board.

**Open Practices:** Experiment 1 disclosures. Preregistration: No aspects of the study were preregistered. Materials: All study materials are publicly available (https://osf.io/n3zhx). Data: All primary data are publicly available (https://osf.io/jk5gw). Analysis scripts: All analysis scripts are publicly available (https://osf.io/x4ptb). Computational reproducibility: The computational reproducibility of the results has been independently confirmed by the journal’s STAR team. Experiment 2 disclosures. Preregistration: No aspects of the study were preregistered. Materials: All study materials are publicly available (https://app.gorilla.sc/openmaterials/792863). Data: All primary data are publicly available (https://osf.io/sra98). Analysis scripts: All analysis scripts are publicly available (https://osf.io/56rqk). Computational reproducibility: The computational reproducibility of the results has been independently confirmed by the journal’s STAR team.

## Notes

1. Performance did not differ systematically with sham feedback (see the Supplemental Results in the Supplemental Material).
