# Supplementary material for: Introspective Access or Retrospective Inference? Mind-Wandering Reports Are Shaped by Performance Feedback
Source: Psychol Sci. 2025 Jun 30;36(7):545–58. doi: 10.1177/09567976251349816 (PMC13428873; doi:10.1177/09567976251349816)
Supplement: sj-pdf-1-pss-10.1177_09567976251349816 – Supplemental material for Introspective Access or Retrospective Inference? Mind-Wandering Reports Are Shaped by Performance Feedback [file sj-pdf-1-pss-10.1177_09567976251349816.pdf]

Supplemental Materials

Introspective access or retrospective inference? Mind wandering reports are shaped by performance feedback

Naya Polychroni[1,2,a], Mahiko Konishi[3], Isa Steinecker[4], & Devin B. Terhune [1,5]

[1] Department of Psychology, Goldsmiths, University of London, London, UK

[2] Department of Experimental Psychology, University of Oxford, Oxford, United Kingdom

[3] Laboratoire de Sciences Cognitives et de Psycholinguistique, Department d'EtudesCognitives, ENS, PSL University, EHESS, CNRS, Paris

[4] Bernstein Center for Computational Neuroscience (BCCN), Berlin, Germany

[5] Department of Psychology, Institute of Psychiatry, Psychology & Neuroscience, King's College London, London, UK

[a] Corresponding author:
Naya Polychroni
naya.polychroni@psy.ox.ac.uk
Department of Experimental Psychology,
University of Oxford
Anna Watts Building, Radcliffe Observatory Quarter,
Woodstock Road, Oxford OX2 6GG, United Kingdom

Experiment 1

Supplemental Methods

Post-task questionnaire

Participants answered a post-task questionnaire that was administered by the experimenter (using pen and paper) in order to gauge participants' awareness of the manipulation (1: "Did you find anything unusual about the task?"; 2: "Did you find anything unusual about the feedback?"; 3: "Was the feedback helpful?"; 4: "How often (%) were you paying attention to the feedback?"; 5: "How often (%) do you think the feedback was accurate?" ; 6: "How often (%) do you think the green feedback was accurate?" ; 7: "How often (%) do you think the red feedback was accurate?"). Participants were then fully debriefed about the manipulation by the experimenter.

Supplemental Results

Evaluating the effect of Sham Feedback on performance (RRTV)

Before our main analysis, we conducted a sensitivity analysis to examine whether performance differed systematically with Sham Feedback, even though it was random and was presented at the end of the sequence of trials. The results confirmed that the manipulation of Sham Feedback did not significantly predict RRTV, B=-.06, SE=.05 [95% CI: -0.17, 0.04], p=.24.

Model Comparisons

In all cases, to test the significance of effects, we used likelihood ratio tests comparing different models against a corresponding larger model (Barr et al., 2013; Meteyard & Davies,

2020). In each case, the nested model excluded the effect of interest (and shared the same random effects with the larger model). The results showed that all predictors significantly improve model fit. We present the results of the model comparisons in **Supplementary Table 1** (Meteyard & Davies, 2020).

Supplementary Table 1.

Model comparisons (likelihood ratio test evaluating significant effects).

**Model name**	**Compared to**	**Fixed effects**	**Random effects**	**LRT**
Predicting ES reports.				
SF+RRTV	-	SF+RRTV	(1|pID) + (SF-1|pID) + (RRTV-1|pID)	
SF only	SF+RRTV	SF	″	12.55***
RRTV only	SF+RRTV	RRTV	″	8.33**
Predicting Confidence in ES reports.				
full	-	ES+SF+SF*ES	(1|pID) + (SF-1|pID) + (ES-1|pID)	
ES+interaction	full	ES+SF*ES	″	13.29***
SF+interaction	full	SF+SF*ES	″	13.56***
SF+ES	full	SF+ES	″	15.32***
Predicting Confidence in on-task reports.				
SF only	-	SF	(1|pID) + (SF-1|pID)	
RE only	SF only	intercept	″	9.35**
Predicting Confidence in MA reports.				
MA	-	MA	(1|pID) + (MA-1|pID)	
RE only	MA	intercept	″	7.19**

Note. LRT=likelihood ratio test, SF= Sham Feedback, RRTV= Rhythmic response times variance, pID=Participant ID, ES=experiential state reports, MA= meta-awareness reports, RE=random effects.

***$p<.001$, **$p<.01$

Extended models

For completion, in addition to the main analyses (see main text), we extended the models to include RRTV and interactions with RRTV as predictors: RRTV×Sham Feedback did not significantly predict either ES, *B*=-.01, *SE*=.05 [95% CI: -0.11, 0.10], *p*=.90, or MA reports, *B*=-.11, *SE*=.08 [95% CI: -0.27, 0.05], *p*=.19. Next, extending the model of confidence in ES reports also did not yield significant effects for either RRTV, *B*=-.41, *SE*=.34 [95% CI: -1.07, 0.25], *p*=.23, or the RRTV×ES interaction, *B*=.55, *SE*=.50 [95% CI: -0.44, 1.53], *p*=.28. Finally, RRTV×MA was not a significant predictor of confidence in MA reports, *B*=0.08, *SE*=1.10 [95% CI: -2.08, 2.23], *p*=.95, whereas RRTV was significant, *B*=1.14, *SE*=.54 [95% CI: 0.08, 2.20], likelihood ratio test: $\chi^2(1)$ =4.44, *p*=.035, such that unaware MW reports increased with greater RRTV.

Experiment 2

Supplemental Methods

Post-task questionnaire

After completing the task, participants answered a post-task questionnaire which assessed awareness of the manipulation (1: “Did you find anything unusual about the task?” (yes, no, prefer not to say); 2: “Did you ever notice the square appearing out of rhythm (earlier or later)?” (yes, no, prefer not to say). They were also provided with a response box for each question. Participants were then fully debriefed regarding the aims of the study. The entire experiment lasted approximately 50 minutes.

Supplemental Results

Model Comparisons

We evaluated the significance of effects using likelihood ratio tests (Barr et al., 2013; Meteyard & Davies, 2020) as in Experiment 1 (**Supplementary Table 2**).

Supplementary Table 2.

Model comparisons (likelihood ratio test evaluating significant effects).

Model name	Compared to	Fixed effects	Random effects	LRT
Predicting ES reports.				
Condition+RRTV	-	Condition + RRTV	(1|pID) + (RRTV-1|pID)	
RRTV only	Condition+ RRTV	RRTV	″	14.38***
Condition only	Condition+ RRTV	Condition	″	56.13***
Predicting MA reports.				
Condition	-	Condition	(1|pID)	
RE only	Condition	intercept	″	4.08*
Predicting Confidence in ES reports.				
full	-	Condition + ES + Condition*ES	(1|pID) + (ES-1|pID)	
ES+interaction	full	ES + Condition*ES	″	8.05***
Condition + ES	full	Condition + ES	″	8.97***
Predicting Confidence in on-task reports.				
Condition	-	Condition	(1|pID)	
RE only	Condition	intercept	″	7.72**
Predicting Confidence in MA reports.				
MA	-	MA	(1|pID) + (MA-1|pID)	

RE only	MA	intercept	″	37.40**

Note. LRT=likelihood ratio test, RRTV= Rhythmic response times variance, pID=Participant ID, ES=experiential state reports, MA= meta-awareness reports, RE=random effects.

****p*<.001, **p<.01, *p<.05

Extended models

For completion, after our main analyses (see main text), we extended models to include RRTV and interactions with RRTV as predictors: RRTV×Condition did not significantly predict ES, *B*=.02, *SE*=.04 [95% CI: -0.05, 0.09], *p*=.60, nor MA reports, *B*=-.03, *SE*=.04 [95% CI: -0.11, 0.05], *p*=.46. Next, RRTV, *B*=.04, *SE*=.13 [95% CI: -0.22, 0.29], *p*=.77, did not significantly predict ES report confidence, whereas the RRTV×ES interaction was a significant predictor, *B*=.27, *SE*=.13 [95% CI: 0.02, 0.51], likelihood ratio test: $\chi^2(1) = 4.53$, *p*=.033. Finally, both RRTV, *B*=.001, *SE*=.20 [95% CI: -0.40, 0.40], *p*=.99, and RRTV×MA, *B*=-.01, *SE*=.32 [95% CI: -0.64, 0.63], *p*=.98, did not significantly predict confidence in MA reports.

Sensitivity analyses

A series of sensitivity analyses were conducted in order to replicate the primary results after excluding participants with atypical response patterns. Analyses of MA reports and MA report confidence were repeated with a reduced sample of 91 participants that had at least 2 instances of each MA report (aware and unaware MW) resulting in 2,679 trials: *M*=29.44, *SD*=13.48, range:5-58; *n*=91. All analyses were also repeated with the conventional five-trial RRTV measure, and after removing trials and participants with skipped delays.

Analysis with the conventional measure of rhythmic response times variance (RRTV).

In this study, RRTV on four trials (*n*-5 to *n*-2 trials before each probe) was used as a predictor. We repeated all analyses which included RRTV as a predictor using RRTV on five trials (*n*-5 to *n*-1 trials before each probe) to align with previous studies using the vMRT (Laflamme et al., 2018). Overall, analysis including the conventional five-trial measure yielded comparable results.

As in the main analysis, RRTV was higher for MW reports, B=.17, SE=.02 [95% CI: 0.13, 0.21], (likelihood ratio test: $\chi^2(1)$ =48.26, p<.001), and participants were more likely to report MW after delay than control trials, B=.17, SE=.05 [95% CI: 0.07, 0.27], (likelihood ratio test: $\chi^2(1)$ =10.06, p=.002). The RRTV×Condition interaction was not significant, B=-.05, SE=.04 [95% CI: -0.13, 0.03], p=.25. In line with the main results, neither RRTV, B=.02, SE=.03 [95% CI: -0.05, 0.09], p=.57, nor the RRTV×Condition significantly predicted MA reports, B=-.02, SE=.05 [95% CI: -0.11, 0.06], p=.59. Condition was significant, B=.17, SE=.08 [95% CI: 0.01, 0.34], p=.042, $\chi^2(1)$ =4.08, p=.043, in line with the main results. Regarding ES report confidence, results were again similar to the main analysis: neither RRTV×ES, B=.22, SE=.13 [95% CI: -0.03, 0.47], p=.082, nor RRTV were significant, B=-.03, SE=.15 [95% CI: -0.32, 0.26], p=.86. Finally, MA report confidence was not significantly predicted from the RRTV×MA interaction, B=-.18, SE=.35 [95% CI: -0.86, 0.51], p=.62, or RRTV, B=-.13, SE=.21 [95% CI: -0.54, 0.28], p=.53.

Analysis with reduced sample of participants.

Analyses for MA reports and MA report confidence were repeated with a reduced sample of 91 participants that had at least 2 instances of each MA report (aware and unaware MW)

resulting in 2,679 trials: M=29.44, SD=13.48, range:5-58; n=91. In contrast to the main results, Condition did not significantly affect meta-awareness reports, B=.16, SE=.09, [95% CI: -0.004, 0.33], p=.056. Both RRTV, B=.02, SE=.03, [95% CI: -0.04, 0.08], p=.55, and RRTV×Condition, B=-.03, SE=.04, [95% CI: -0.11, 0.05], p=.46, remained non-significant. The results for MA report confidence were comparable: Condition was marginally non-significant, B=1.35, SE=.70, [95% CI: -0.02, 2.72], p=.053, and the Condition×MA interaction was non-significant, B=-1.57, SE=1.09, [95% CI: -3.71, 0.58], p=.15, whereas MA remained significant, B=-8.32, SE=1.38, [95% CI: -11.03, -5.62] (likelihood ratio test: $\chi^2(1)$ =30.57, p<.001). Neither the RRTV×MA interaction, B=-.03, SE=.33, [95% CI: -0.68, 0.62], p=.93, nor RRTV were significant, B=-.06, SE=.22, [95% CI: -0.49, 0.37], p=.79.

References

Barr, D. J., Levy, R., Scheepers, C., & Tily, H. J. (2013). Random effects structure for confirmatory hypothesis testing: Keep it maximal. *Journal of Memory and Language*, *68*(3), 255–278. https://doi.org/https://doi.org/10.1016/j.jml.2012.11.001

Laflamme, P., Seli, P., & Smilek, D. (2018). Validating a visual version of the metronome response task. *Behavior Research Methods 50*(4), 1503–1514. https://doi.org/10.3758/s13428-018-1020-0

Meteyard, L., & Davies, R. A. I. (2020). Best practice guidance for linear mixed-effects models in psychological science. *Journal of Memory and Language*, *112*, 104092. https://doi.org/https://doi.org/10.1016/j.jml.2020.104092
